# New Zealanders living with a family member who has a long-term health condition: cross sectional analysis of integrated Census and administrative data

**DOI:** 10.23889/ijpds.v9i5.2764

**Published:** 2024-09-18

**Authors:** Lisa Underwood, Nick Bowden, Andrea Teng, Ofa Dewes, Lukas Marek, Barry Milne

**Affiliations:** 1 University of Auckland; 2 University of Otago; 3 Tongan Health Society; 4 University of Canterbury

## Background

Living with a family member who has a long-term health condition (HC) is associated with poorer health and well-being outcomes, but the number and socio-demographics of people and families impacted by a family member who has an HC is unknown.

## Methods

Using the Integrated Data Infrastructure (IDI), a collection of linked administrative datasets for the full New Zealand (NZ) population, we identified n= 1,043,172 families using 2013 NZ Census data, and used health data over the previous 5-years to ascertain whether people in these families (n=3,137,517) had cancer, chronic obstructive pulmonary disease, coronary health disease, diabetes, dementia, gout, stroke, traumatic brain injury (TBI) or a mental health/behavioural condition (MHBC).

## Results

Over 60% of families included at least one person with a HC. The most common HCs were MHBCs (39.4% of families), diabetes (16.0%), and TBI (13.9%). A high proportion of multi-generation families (73.9%) included a member with a HC. Two-thirds (67.7%) of Pacific Peoples either had a HC themselves or were living with a family member who had a HC, compared with 62.3% of Europeans and 62.5% of Māori (the indigenous peoples of NZ). At the highest level of socioeconomic deprivation, 57.6% of children lived with a family member who had a HC.

## Conclusions

Three in five NZ families were living with a HC, with differences in the proportion affected according to family composition, socio-economic status and an individuals’ ethnicity. This suggests that there are a substantial number of people at risk of the associated poor outcomes.

